# Development of Alternative Porous Magnesium Potassium Phosphate Cements as Thermal Insulating Materials

**DOI:** 10.3390/ma18173946

**Published:** 2025-08-22

**Authors:** Jessica Giro-Paloma, Jofre Mañosa, Alex Maldonado-Alameda, Anna Alfocea-Roig, Sergio Huete-Hernández, Josep Maria Chimenos, Joan Formosa

**Affiliations:** 1 Departament de Ciència de Materials i Química Física, Universitat de Barcelona, Martí i Franquès, 1, 08028 Barcelona, Spain; jessicagiro@ub.edu (J.G.-P.); jofremanosa@ub.edu (J.M.); annaalfocea@ub.edu (A.A.-R.); 2 Fundación Centro Tecnológico de Investigación Multisectorial (CETIM), Parque Empresarial de Alvedro, 20, 15180 Culleredo, Spain; amaldonado@cetim.es; 3 Departament d’Enginyeria Civil i Ambiental, Universitat Politècnica de Catalunya, Jordi Girona, 1-3, 08034 Barcelona, Spain; sergio.huete.hernandez@upc.edu

**Keywords:** MKPC, hydrogen peroxide, porosity, thermal insulation, thermal conductivity

## Abstract

Magnesium potassium phosphate cement (MKPC), a type of chemically bonded phosphate ceramic (CBPC), presents a promising alternative to ordinary Portland cement (OPC). This study focuses on developing sustainable MKPC (sust-MKPC) as a thermally passive material for building applications. A low-grade magnesium oxide (LG-MgO) industrial by-product was utilized to formulate sust-MKPC, with hydrogen peroxide employed as an air-entraining agent (AEA) to induce high porosity and enhance thermal insulation while supporting sustainability goals by reducing energy consumption in climate control systems. Seven formulations incorporating varying hydrogen peroxide contents (0, 1, 2, 3, 5, 7.5, and 10 wt.%) were prepared to evaluate the impact of AEA on the thermal and physicomechanical properties. Comprehensive characterization, including porosity and thermal conductivity measurements, revealed that increasing the AEA content significantly improved thermal inertia and lowered thermal conductivity due to porosity. However, this enhancement was accompanied by a marked reduction in mechanical strength and density, highlighting the trade-off between thermal performance and structural integrity in porous sust-MKPC formulations.

## 1. Introduction

In recent years, the rapid expansion of industrial activities has significantly increased global energy demand and the depletion of non-renewable natural resources. This trend has raised critical environmental concerns, particularly regarding the exponential growth of pollution and waste associated with the extraction, processing, and production of materials. In response, governments and industries are under mounting pressure to adopt more sustainable practices. Transitioning toward a sustainable economic and societal model requires the implementation of comprehensive strategies aimed at minimizing environmental impact and energy consumption, notably through the reduction of CO_2_ emissions and other greenhouse gases (GHGs). To achieve these goals, it is imperative to promote the recycling, revalorization, and reuse of waste, by-products, and end-of-life materials [1]. The cement industry is investigating alternative raw materials and fuels to reduce GHGs [2]. The use of industrial by-products in construction [3] and the development of new materials can lower CO_2_ emissions and enhance building energy efficiency [4]. Durability remains a key consideration in adopting these alternative binders [5]. Despite extensive data on Portland cement production [6], there is a clear need for sustainable binders capable of competing in terms of environmental performance [7].

Among these, chemically bonded phosphate ceramics (CBPCs), and specifically magnesium phosphate cements (MPC), have garnered increasing attention. MPCs are typically synthesized from magnesium oxide (MgO), which acts as the basic component, and an acidic phosphate source. The preparation of MPC varies depending on the specific raw materials employed. For instance, when ammonium dihydrogen phosphate (ADP) is used, ammonia is released into the environment during the reaction. In contrast, monopotassium phosphate (MKP) is a preferred alternative, as it eliminates the need for retarders and offers additional processing advantages [8]. Although MKP is generally less economical than other phosphate sources, it is widely adopted due to its beneficial properties, including lower solubility, improved workability, and slower hardening kinetics, which facilitate handling and casting [9]. However, an excessive amount of MKP can detrimentally affect the mechanical performance of the final product [10]. The MgO required for magnesium potassium phosphate cement (MKPC) synthesis can be derived from various sources, including thermal decarbonation of magnesite (MgCO_3_) or dolomite (CaMg(CO_3_)_2_), as well as from seawater-derived magnesium chloride (MgCl_2_) via electrolysis. To achieve the appropriate level of reactivity for MKPC formation, MgO must undergo thermal treatment, typically at 1500 °C for 5 h, to produce dead-burned MgO [8]. In this study, a sustainable MKPC (sust-MKPC) was developed using a low-grade MgO (LG-MgO) obtained as a by-product from the air pollution control system of an industrial magnesite calcination process. This LG-MgO is a dead-burned material containing approximately 50–60 wt.% MgO, along with inert phases that did not fully decompose during processing, such as residual MgCO_3_, CaMg(CO_3_)_2_, and CaCO_3_ [11].

The exothermic acid–base reaction between phosphate sources and MgO in aqueous media proceeds slowly enough to allow phosphate gel formation, yielding K-struvite (KMgPO_4_·6H_2_O) as the primary crystalline product [12]. MKPC also contains an amorphous phase that contributes to its distinctive microstructure [13]. This dual-phase composition distinguishes MKPC from conventional cements and supports its use as a matrix for reinforcing agents. MKPC offers excellent thermal stability, chemical resistance, and long-term durability under aggressive conditions [14]. Furthermore, MKPC is a non-toxic, inorganic cement. Due to its phosphate-based composition, it also shows high biocompatibility, making it suitable for biomedical and environmental applications [15].

Given the advantageous properties of MKPC, its application has expanded into diverse fields, including its use as a structural repair material for bridges, roads, and airport pavements [16,17]. Moreover, MKPC has shown significant potential in the nuclear industry, particularly for the stabilization and encapsulation of radioactive and hazardous waste, due to its chemical durability and containment capabilities [18,19]. Beyond their practical applications, ongoing research into CBPCs and MPCs is also driven by their environmental benefits compared to OPC. For instance, the production of one ton of OPC typically results in approximately 900 kg of CO_2_ emissions and requires around 5 GJ of energy, whereas MPC production can reduce CO_2_ emissions by up to 40% for the same amount of material [20]. Nevertheless, a recent study indicates that the production of one ton of MKPC results in 1160 kg of CO_2_-eq. However, if the source of MgO is a secondary resource, the emissions are significantly reduced to 430 kg of CO_2_-eq. This means that the production-related emissions of MKPC can be reduced by more than 60% when secondary resources are used as the raw material for MgO, and by nearly 50% when compared to the emissions associated with producing 1 ton of OPC [21]. The use of LG-MgO further enhances the environmental credentials of MKPC. As a by-product of the magnesite calcination process, LG-MgO not only diverts waste from industrial processes but also eliminates the need for high-temperature thermal treatment, thereby reducing energy consumption and associated emissions. Consequently, the development of a sustainable MKPC (sust-MKPC) aligns closely with the European Union’s environmental and sustainability goals by promoting circular resource use and mitigating high energy demands and pollution.

In addition to reducing the carbon footprint of construction materials, this study seeks to enhance the energy efficiency of buildings by minimizing reliance on heating, ventilation, and air conditioning (HVAC) systems to maintain thermal comfort. Specifically, the focus is on developing a sust-MKPC with tailored porosity that can contribute to passive thermal regulation. By optimizing its porous structure, the proposed material aims to reduce the operational energy demand of buildings, thereby lowering both CO_2_ emissions and total energy consumption.

To develop sust-MKPC with thermal insulation properties from an LG-MgO by-product while meeting environmental sustainability criteria, an air-entraining agent (AEA) is typically used to reduce the thermal conductivity of the sust-MKPC matrix [22]. A wide range of foaming agents is commonly employed in porous cementitious systems, including surfactants, proteins, synthetic compounds, metal powders, and hydrogen peroxide (H_2_O_2_) [14]. Among these, H_2_O_2_ is widely used due to its effectiveness as a chemical pore-forming agent. It is an unstable compound that decomposes exothermically into oxygen and water, thereby generating gas bubbles that contribute to pore formation within the cement matrix. The decomposition rate of H_2_O_2_ is strongly influenced by both temperature and pH, increasing under higher values of either parameter [23]. With respect to pH, H_2_O_2_ behaves as a weak acid and undergoes ionization according to Equation (1). In alkaline conditions, it further reacts as shown in Equation (2), while under strongly alkaline environments, it can decompose rapidly through the reaction described in Equation (3). These pH-dependent reactions are critical in controlling the gas generation rate, and thus the porosity and microstructure of the final sust-MKPC composite.

(1) $$H_{2}O_{2(l)}↔H_{\left(aq.\right)}^{+}+{OOH}_{\left(aq.\right)}^{-}$$

(2) $$H_{2}O_{2(l)}+{OH}_{\left(aq.\right)}^{-}↔H_{2}O_{\left(l\right)}+{OOH}_{\left(aq.\right)}^{-}$$

(3) $$H_{2}O_{2(l)}↔H_{2}O_{\left(l\right)}+\frac{1}{2}O_{2(g)}$$

As temperature increases, so does the decomposition rate of hydrogen peroxide (H_2_O_2_), along with the pH of the surrounding medium. For optimal stability, H_2_O_2_ requires a pH below 4.5. However, when the pH exceeds five, its decomposition accelerates significantly. Moreover, the presence of impurities, particularly transition metals such as Fe, Cu, Mn, Ni, and Cr, can strongly catalyze the decomposition reaction. In the context of formulating a high-porosity sust-MKPC for thermal insulation panels, the rapid decomposition of H_2_O_2_ is desirable, as it promotes effective pore formation. Notably, the use of LG-MgO as the magnesium source inherently contributes to three key factors that enhance the decomposition kinetics of H_2_O_2_: (i) the initial pH of the system is highly alkaline due to the solubility equilibrium of MgO, though it becomes near-neutral upon completion of the MKPC setting reaction; (ii) an exothermic neutralization reaction between MKPC precursors results in a notable increase in temperature; (iii) the LG-MgO by-product contains catalytically active metal ions such as Fe and Mn, which further promote H_2_O_2_ decomposition. Collectively, these conditions facilitate the controlled decomposition of H_2_O_2_, leading to the formation of a porous structure in the sust-MKPC matrix, an essential feature for improving its thermal insulation performance.

The primary objective of this research is to evaluate the potential of H_2_O_2_ as a pore-forming agent in sust-MKPC formulations, aiming to enhance their thermal insulation properties for use in passive conditioning systems. The use of LG-MgO, an industrial by-product, aligns with key environmental sustainability criteria by promoting waste valorization, reducing the extraction of natural MgO resources, and lowering the CO_2_ emissions associated with cement production. Given that critical material properties such as mechanical strength, permeability, diffusivity, and shrinkage are closely linked to porosity and pore size distribution, various H_2_O_2_ dosages were explored to optimize the balance between thermal and mechanical performance. Accordingly, a series of sust-MKPC formulations was developed incorporating H_2_O_2_ as an AEA, with the goal of decreasing thermal conductivity while preserving structural integrity.

## 2. Materials and Methods

### 2.1. Materials

LG-MgO is a brown particulate material classified as a dead-burned magnesia by-product derived from the thermal treatment of natural magnesite (MgCO_3_) in rotary kilns. Approximately 25 kg of LG-MgO were collected from multiple stockpiles, supplied by Magnesitas Navarras, S.A. (Navarra, Spain). The incorporation of LG-MgO in the formulation of sust-MKPC offers both economic and environmental benefits. Its use reduces the reliance on virgin mineral extraction, lowers the raw material cost of MKPC, and contributes to the valorization of industrial waste streams.

The second essential precursor for sust-MKPC synthesis was mono-potassium phosphate (KH_2_PO_4_, commonly referred to as MKP), a white, water-soluble salt widely used as an agricultural fertilizer. The MKP employed in this study was supplied by Rotem (Israel). As a food-grade product, MKP is not classified as a hazardous chemical, contributing to the sustainability and cost-effectiveness of the developed sust-MKPC formulations.

### 2.2. Methods

#### 2.2.1. Sust-MKPC Preparation

The proportions of the sust-MKPC formulations are compiled in Table 1. Initially, the dry components, LG-MgO and MKP, were weighed and pre-mixed in a laboratory mixer for 40 s to ensure homogeneity. The total solid mass (1800 g) consisted of 60 wt.% LG-MgO and 40 wt.% MKP, in line with the authors’ previous optimization studies [24]. Subsequently, the liquid phase, comprising distilled water and hydrogen peroxide (H_2_O_2_), was added. The total liquid content corresponded to a liquid-to-solid (L/S) ratio of 0.34 (610 g). Seven different formulations were prepared with varying H_2_O_2_ contents: 0, 1, 2, 3, 5, 7.5, and 10 wt.%, relative to the total liquid mass. The percentages of water and hydrogen peroxide in Table 1 are relative to the total solid (LG-MgO and MKP) content.

The reaction began immediately upon the initial contact between the liquid and dry components. Mixing was carried out in two stages: 20 s at low speed followed by 90 s at high speed. The fresh paste was then poured into wooden molds with internal dimensions of 150 × 150 × 40 mm^3^ and then subjected to vibration for 10 s to eliminate initially entrapped air and level the surface. Hence, any resulting porosity determined thereafter can be predominantly ascribed to the decomposition of H_2_O_2_. Each mold was fitted with a heavy cover to ensure a uniform top surface. A thermocouple was embedded and securely fixed in each mold to monitor the temperature profile throughout the reaction and setting process. After 24 h, the specimens were demolded, weighed, and cured under ambient laboratory conditions (22 ± 2 °C; 50 ± 5% of relative humidity (R.H.)). Following a three-week curing period, the samples were transferred to a silica gel chamber to undergo controlled drying at low humidity (below 20% of R.H.), which also helped to stabilize their dimensions. Just prior to mechanical testing (after 28 days of curing), all specimens were trimmed to uniform dimensions of 150 × 40 × *h* mm^3^. This step was necessary because the presence of the H_2_O_2_ caused noticeable variations in the final sample height (*h*).

#### 2.2.2. LG-MgO Characterization

The particle size distribution (PSD) of LG-MgO was determined by dispersing the powder in acetone and analyzing it using a Beckman Coulter^®^ LS 13 320 laser diffraction particle size analyzer. Semi-quantitative elemental composition of the LG-MgO powder was obtained via X-ray fluorescence (XRF) spectroscopy using a Panalytical Philips PW 2400 sequential XRF spectrometer equipped with UniQuant^®^ V5.0 software for data analysis. Crystalline phase identification was performed by X-ray diffraction (XRD) using a Siemens D-500 powder diffractometer operating in Bragg–Brentano geometry with CuK_α_ radiation. The specific surface area and porosity-related characteristics of LG-MgO powders were evaluated using the Brunauer–Emmett–Teller (BET) method on a Micromeritics Tristar 3000 analyzer, providing insight into the material’s reactivity via the total active surface area.

#### 2.2.3. Sust-MKPC Characterization

To investigate the molecular structure and chemical bonding of the sust-MKPC formulations, Fourier transform infrared (FT-IR) spectroscopy was conducted employing the attenuated total reflectance (ATR) sampling technique. Measurements were carried out on a Perkin Elmer Spectrum Two™ spectrometer, equipped with a Dynascan™ interferometer and OpticsGuard™ technology. The spectral range was set between 4000 and 500 cm^−1^ with a resolution of 0.5 cm^−1^.

The setting behavior of the sust-MKPC was assessed by monitoring the temperature evolution during the early curing stage. A data logger continuously recorded the temperature profiles, which reflect the exothermic chemical reactions and the associated physical transformations occurring during the hardening process.

The apparent density (ρ_app_), relative density (ρ_rel_), and porosity of the sust-MKPC samples were determined in triplicate following the UNE-EN 1015-10 standard, which is based on Archimedes’ principle. This methodology was used to assess the influence of the AEA on the physical properties of the different formulations.

Porosity was also determined through image analysis using ImageJ software (2015). Two-dimensional cross-sectional images were digitized and processed to distinguish between porous and solid regions by quantifying the number of pore pixels. Image noise was carefully removed to ensure accurate segmentation and analysis.

The thermal behavior of the sust-MKPC samples was evaluated in triplicate through a series of tests, including thermal cycling, thermal conductivity, heat capacity, and thermal diffusivity measurements. Thermal conductivity and diffusivity were measured after 28 days of curing using a Quickline TM-30 thermal conductometer (Anter Corporation), which operates according to the ASTM D5930 standard. This instrument employs a dynamic measurement method by applying thermal pulses through a probe, allowing for the determination of thermal conductivity within the range of 0.1 to 2.0 W·m^−^^1^·K^−^^1^. Thermal diffusivity (α) characterizes the rate at which a material reaches thermal equilibrium, while thermal conductivity (λ) describes its ability to conduct heat. In a homogeneous material, α and λ are related through the material’s density (ρ) and specific heat capacity (C_p_), according to Equation (4).

(4) λ = α · C_p_ · ρ

The thermal cycling test aimed to assess the material’s response to temperature changes and the time required to reach thermal equilibrium. For this test, a thermocouple was embedded inside each specimen by drilling a small cavity to monitor the internal temperature. Additional thermocouples were placed to monitor the ambient temperature and record the surface temperature variation on one external face of the specimen throughout the thermal cycles. Initially, the samples were subjected to controlled heating and cooling cycles. They were first conditioned in a refrigerator at 10 °C for several hours until thermal equilibrium was reached. Subsequently, the samples were transferred to an oven set at 40 °C for 3 h, followed by a return to the refrigerator for 24 h.

The elastic modulus (MOE), flexural strength, and compressive strength of the sust-MKPC samples were evaluated in triplicate after 28 days of curing. The MOE measurements were conducted following the UNE-EN ISO 12680-1 standard, which specifies the test methods for refractory products. Since this method is applicable to isotropic and homogeneous material systems, the results are not considered absolute values but are instead used as control parameters to enable comparison with mechanical strength results and to support the porosity measurements obtained. The pulse velocity (in m·s^−1^) was calculated using Equation (5), which relates the fundamental resonance frequency obtained by applying an impulse to each sample and the wavelength of the first harmonic. In this equation, *L* represents the length of the specimen (in m), and *f* denotes the longitudinal vibration frequency (in Hz). The elastic modulus (MOE) was then derived based on this pulse velocity, linking the dynamic mechanical response of the material to its elastic properties.

(5) *V* = *2*·*L*·*f*

Once the longitudinal pulse velocity was determined, the elastic modulus (MOE) for each formulation was calculated using the material’s density (ρ, kg·m^−^^3^) according to Equation (6).

(6) *MOE* = ρ·*V^2^*

Subsequently, the flexural (σ_f_) and compressive (σ_c_) strengths were determined in accordance with the UNE-EN 196-1 standard. Flexural strength tests were performed using an Incotecnic MULTI-R1 device, applying a load at a rate of 5 kg·s^−1^ with a crosshead speed of 10 mm·min^−1^ until specimen failure. After the flexural test, the two halves of each specimen were used for compression testing, where a load was applied at 240 kg·s^−1^ until fracture occurred.

## 3. Results and Discussion

### 3.1. Physico-Chemical Characterization of LG-MgO

**Figure 1:**
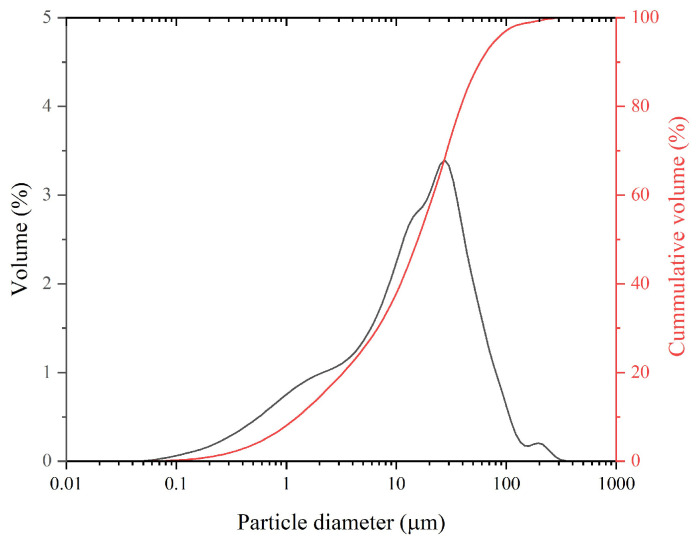
Particle size distribution of LG-MgO.

The particle size of the powder is a critical parameter, as it can significantly influence the reaction rate of MKPC formation. In general, larger particle sizes are associated with slower reaction kinetics [25]. Figure 1 shows the particle size distribution of LG-MgO, which exhibits a mean particle size (d_50_) of 25.13 μm.

The chemical composition of LG-MgO, determined by XRF analysis, is presented in Table 2, which describes the most stable oxides of each element detected in the sample. As expected, Mg was the major element, followed by Ca, S, Si, Fe, and Al oxides with lower percentages. The 10% loss on ignition (LOI) was attributable to the possible carbonates and hydrated phases in LG-MgO.

To complement the XRF analysis, the crystallographic mineral phases were identified by XRD. The LG-MgO diffractogram revealed periclase (MgO) as the predominant crystalline phase, resulting from the decarbonization of natural magnesite during the thermal process. Several minor phases were also identified, including calcite (CaCO_3_), dolomite (CaMg (CO_3_)_2_), quartz (SiO_2_), magnesite (MgCO_3_), anhydrite (CaSO_4_), brucite (Mg(OH)_2_), and calcium oxide (CaO).

The specific surface area of LG-MgO was evaluated through a BET analyzer, yielding a value of 5.2 m^2^·g^−1^. In comparison with other commercial caustic magnesia products (e.g., 70 m^2^·g^−1^), this significantly lower value corroborates the reduced reactivity of the LG-MgO by-product.

### 3.2. FT-IR

**Figure 2:**
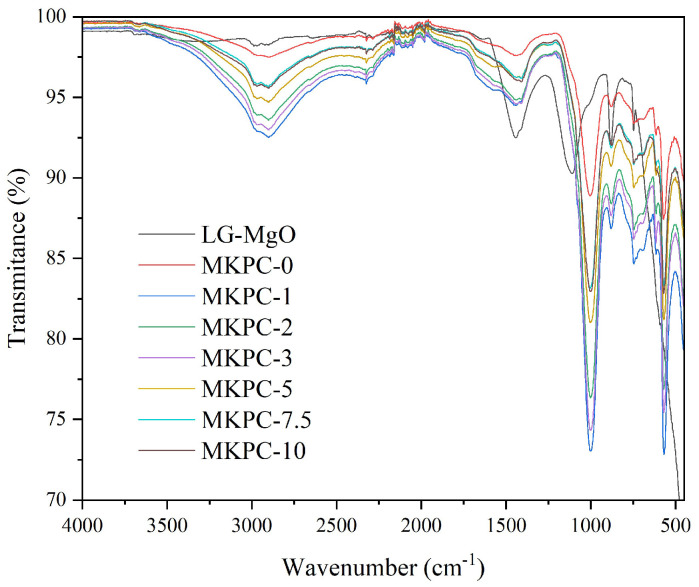
FT-IR spectra of LG-MgO and sust-MKPC.

The most relevant peak in the LG-MgO spectrum was observed at 740 cm^−1^, corresponding to the Mg-O bond. The compositional differences among the various sust-MKPC formulations were analyzed and are presented in Figure 2. The spectrum of MKPC-0 primarily exhibited the characteristic bands of K-struvite [26], the main crystalline mineral phase in MKPC. All formulations showed consistent spectral profiles, with no evidence of compositional changes or the formation of new chemical bonds, indicating that K-struvite remained the principal hydration product across all samples.

### 3.3. Setting Time and Temperature

**Figure 3:**
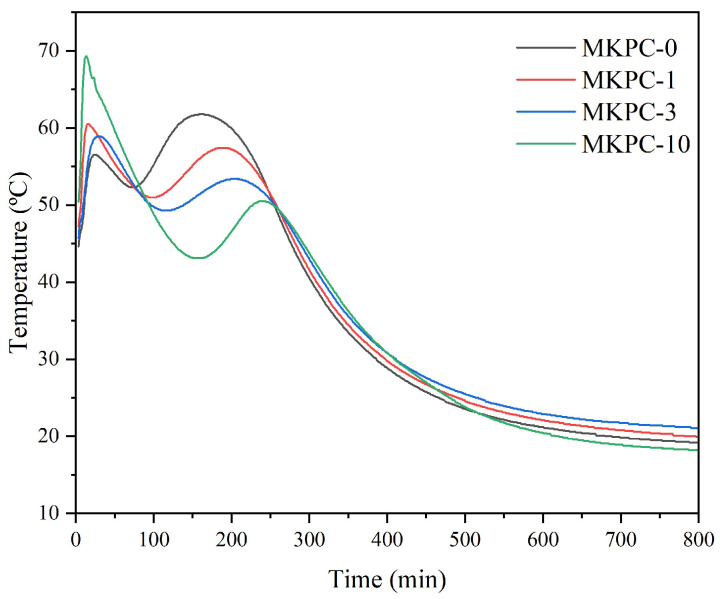
Setting of sust-MKPC samples.

Figure 3 displays the temperature–time profile during the setting of sust-MKPC. The maximum temperature increased with higher H_2_O_2_ content, as the reaction becomes more exothermic due to the decomposition of H_2_O_2_ into H_2_O and O_2_. The first peak can be attributed to the setting time of each sust-MKPC formulation, marking the point at which the fresh paste hardened and lost its plasticity as a result of the chemical reaction between water and the precursors in the mixture. For MKPC-10, the maximum temperature reached 80 °C, while MKPC-0 peaked at approximately 60 °C. After the first sharp exothermic peak, associated with the acid–base reaction (chemical setting reaction), a second broad peak due to cement crystallization is detected. The second peak was delayed with increasing H_2_O_2_ content, also reducing its maximum temperature, suggesting that the incorporation of H_2_O_2_ hinders the formation and crystallization of K-struvite.

### 3.4. Density and Porosity

**Figure 4:**
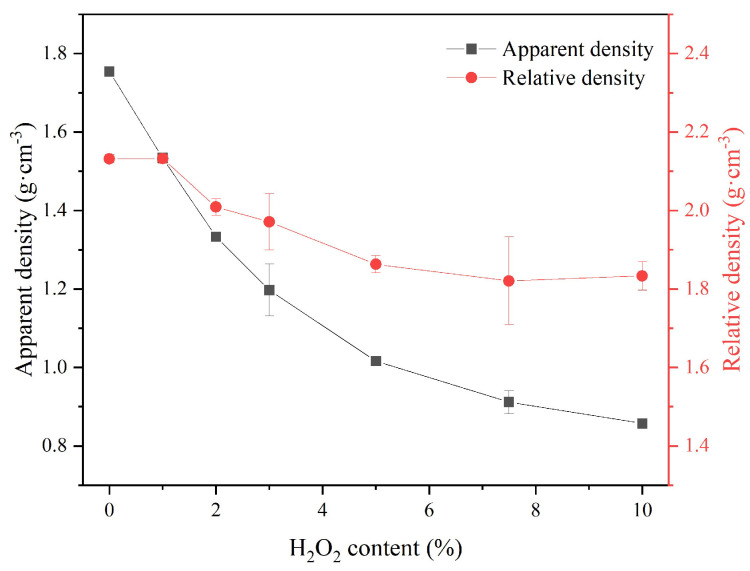
Apparent density (ρ_app_) and relative density (ρ_rel_) results for the sust-MKPC.

As shown in Figure 4, both the apparent density (ρ_app_) and relative density (ρ_rel_) at 28 days significantly decreased with the increasing H_2_O_2_ content. MKPC-10 exhibited values of ρ_app_ as low as 0.8 g·cm^−3^. The densities of the sust-MKPC formulations were significantly lower than that of OPC (3.15 g·cm^−3^), confirming that sust-MKPCs can be considered as lightweight materials.

**Figure 5:**
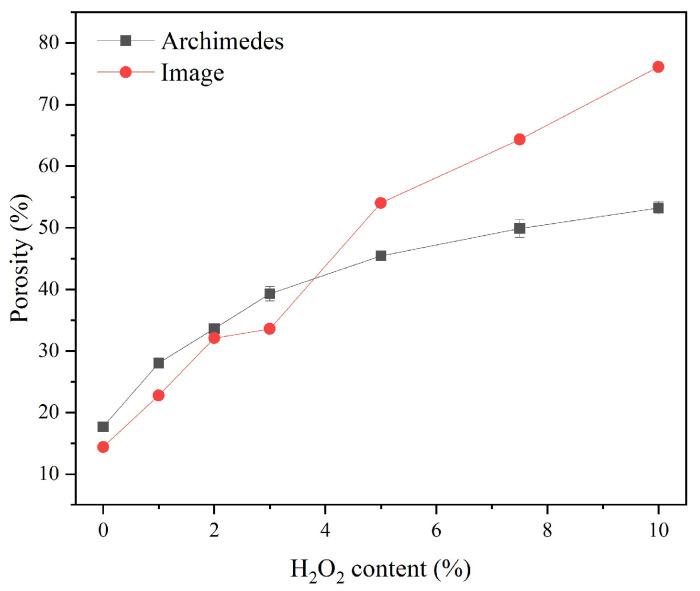
Porosity of sust-MKPC measured through Archimedes’ principle and image treatment.

Figure 5 displays the porosity of the sust-MKPC samples, as determined by the Archimedes principle and image analysis techniques. Porosity increased with the increasing AEA content. This trend can be attributed to the decomposition of H_2_O_2_, which acts as an air-entraining agent and promotes pore formation within the sust-MKPC formulations. Moreover, as seen in Figure 3, the maximum temperature during cement hydration was significantly higher with H_2_O_2_ incorporation, promoting H_2_O evaporation. It is significant to point out that the results were exceptionally high for MKPC-10, as the sample reached over 50% of porosity through Archimedes’ measurements and almost 80% through image analysis. Therefore, considering the high porosity values obtained, formulations exhibiting porosity levels above 40%, corresponding to H_2_O_2_ contents greater than 3 wt.%, can be considered suitable for thermal insulation applications. The observed increase in porosity with higher H_2_O_2_ content also accounts for the significant reduction in the density of the sust-MKPC formulations.

Notably, at low H_2_O_2_ contents, the values of porosity measured through both methods were comparable. However, at high percentages, the porosity measured through image analysis was significantly higher. This discrepancy may be attributed to the presence of enclosed pores, which are not filled with water during Archimedes-based measurements but are observed in a cross-section image of the sample.

**Figure 6:**
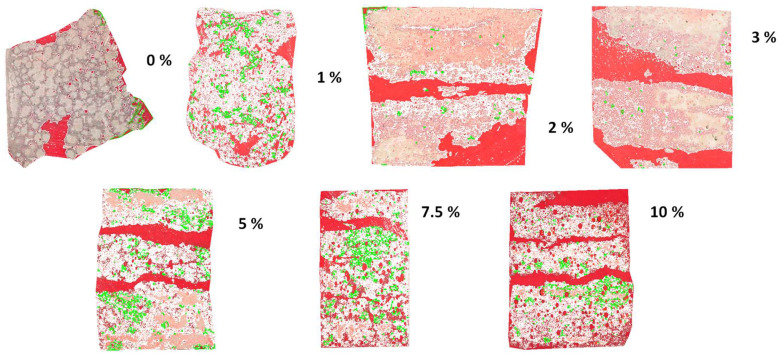
Images treated with ImageJ of the sust-MKPC formulations with increasing H_2_O_2_ content, from MKPC-0 to MKPC-10.

Figure 6 presents 2D cross-section images of sust-MKPC formulations (MKPC-0 to MKPC-10), with a progressively increasing H_2_O_2_ content analyzed using ImageJ software. The corresponding H_2_O_2_ content (wt.%) is indicated for each specimen. For the porosity assessment, the samples were precision-sectioned into thin slices and embedded in red resin, which infiltrated and visually highlighted the pore spaces. It should be noted that the absolute thickness of the thin slices and the size of the image are not considered in this analysis; the images are intended solely to provide a rapid visual comparison of porosity trends as a function of AEA content. In the images, the MKPC matrix appears white, the resin-filled pores appear red, and green outlines have been superimposed to delineate the boundaries of pores with poorly defined edges, thereby improving surface definition and measurement reproducibility. In addition to the overall increase in total porosity observed in Figure 5, Figure 6 reveals a progressive enlargement of pore size with a higher AEA content, culminating in the formation of large cracks. These cracks are likely the result of coalescence between adjacent large pores. This behavior can be partially attributed to the extended setting time, which reduces the viscosity of the binder phases — primarily due to the formation of K-struvite. Lower viscosity decreases the resistance of the paste to internal gas expansion, enabling the growth of larger pores. Additionally, the simultaneous decrease in surface tension amplifies the influence of gas expansion forces within the matrix, thereby promoting pore enlargement and, in some cases, the merging of pores into microcracks.

### 3.5. Thermal Properties

**Figure 7:**
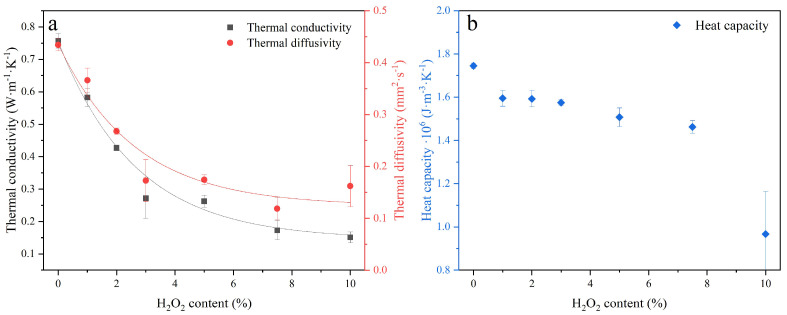
Thermal properties of sust-MKPC as (**a**) thermal conductivity and thermal diffusivity, and (**b**) volumetric heat capacity.

The conductivity, thermal diffusivity, and heat capacity were measured to assess the impact of increased porosity on the insulating properties of sust-MKPC formulations. The thermal conductivity and thermal diffusivity are shown in Figure 7a, and the volumetric heat capacity is plotted in Figure 7b. The increase in AEA content contributed to a significant reduction in the thermal conductivity of the sust-MKPC formulations. This is strongly related to the porosity generated by the decomposition of H_2_O_2,_ triggered by the exothermic reaction in sust-MKPC, as well as the evaporation of the mixing water due to the temperature rise during the hydration of binder phases. Accordingly, the increased porosity in these samples resulted in diminished heat transport, which is expected to lead to lower thermal inertia. The developed sust-MKPC cannot be considered insulating materials since their thermal conductivity exceeds 0.09 W·m^−1^·K^−1^ [27]. However, the thermal conductivity values are substantially lower than conventional building materials, such as OPC [28]. The thermal conductivity values obtained are consistent with those reported in previous studies on MPC-based foamed concretes formulated with H_2_O_2_ as a foaming agent, although using ADP instead of MKP [29].

Since thermal diffusivity represents the rate at which heat propagates through a material, its reduction with increasing H_2_O_2_ content followed a similar exponential trend to that of thermal conductivity. This behavior may be influenced by pore morphology. Specifically, larger pores tend to disrupt heat transfer more significantly than smaller ones, leading to a more pronounced reduction in thermal diffusivity. Moreover, at up to 3 wt.% AEA content, the thermal diffusivity decreases substantially, after which it remains relatively stable.

Regarding the volumetric heat capacity of the different sust-MKPC formulations, it exhibited a generally decreasing trend, with particularly low values observed in the MKPC-10 sample. However, this sample also showed significant variability in the measurements, likely due to its highly porous, microcracked, and irregular structure.

**Figure 8:**
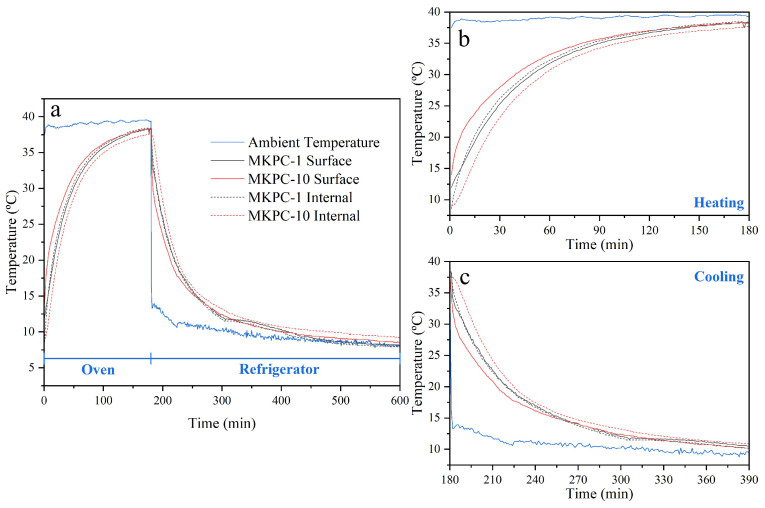
Internal and surface temperature of MKPC-1 and MKPC-10 during thermal cycling in the regions of (**a**) full cycle, (**b**) heating, and (**c**) cooling.

Thermal cycling tests were conducted to evaluate the influence of H_2_O_2_ content, used as AEA, on the thermal inertia of sust-MKPC formulations. Figure 8 depicts the ambient, internal, and surface temperature of representative specimens (MKPC-1 and MKPC-10) during a heating–cooling cycle. The addition of the AEA agent improves the thermal inertia of sust-MKPCs, due to its lower thermal conductivity. Therefore, the higher content of H_2_O_2_ enhanced the gap between the inner and the outer measured temperature, attributed to the higher porosity of the samples. This higher thermal inertia could help maintain a comfortable temperature in buildings.

As observed, the evolution of the internal and surface temperatures in sust-MKPC specimens during the heating–cooling cycle reveals distinct thermal responses associated with their porosity levels. MKPC-10, characterized by a higher content of AEA and consequently greater porosity, exhibits a pronounced temperature gradient between the internal and surface regions at the onset of each thermal transition. In some instances, this gradient exceeds 7 °C. Conversely, MKPC-1, with lower porosity, shows negligible differences between the internal and surface temperatures, indicating a more uniform thermal response. As expected, the temperature gradients observed in MKPC-10 tend to diminish over time as thermal equilibrium is progressively reached within the specimen. This behavior highlights the significant influence of porosity on the thermal inertia of sust-MKPCs, as higher porosity enhances the material’s capacity to delay internal temperature changes and thereby modulates the heat transfer dynamics.

### 3.6. Mechanical Properties

**Figure 9:**
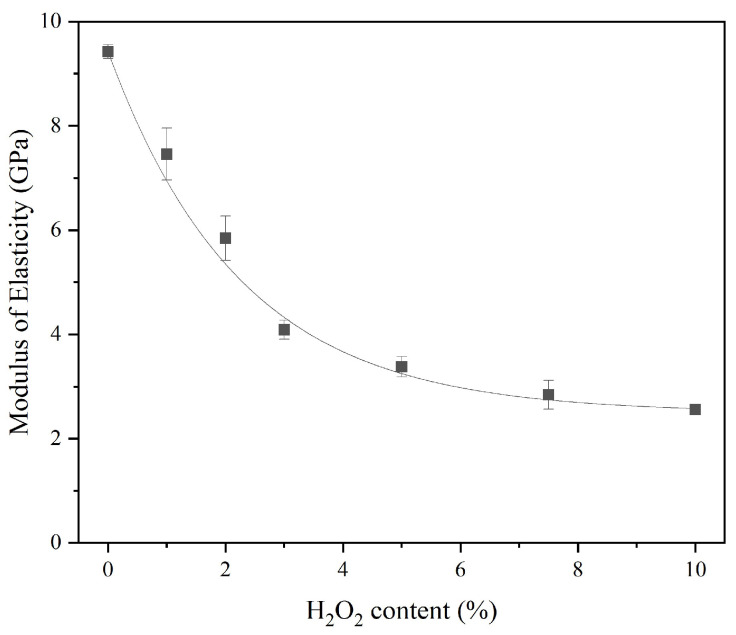
Modulus of elasticity of sust-MKPC.

An increase in porosity leads to a reduction in density, which implies less compactness of the sust-MKPC formulations and results in a lower MOE. Moreover, both ultrasound wave propagation through the material and stiffness decrease as porosity increases [5]. This trend is observed in Figure 9, where an exponential decrease in the MOE of the samples can be observed with an increasing AEA content.

**Figure 10:**
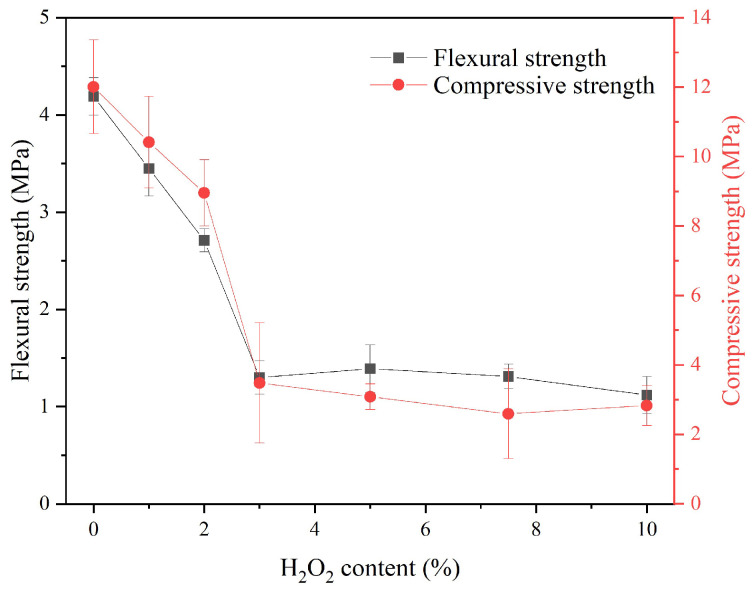
Flexural and compressive strength of sust-MKPC with varying H_2_O_2_ content.

Figure 10 displays the flexural and compressive strength of the sust-MKPC formulations. Similar to the MOE results, the increase in the porosity led to a reduction in the strength of the binder. It is important to emphasize that the samples exhibited cracks caused by the neutralization exothermic reaction between the acid (MKP) and the base (LG-MgO), and significant pore generation (see Figure 6). This formulation exhibited uncontrolled expansion during the rapid setting time. As a result, the samples displayed a decreasing trend in strength with an increasing H_2_O_2_ content, reaching a minimum at 3%. Beyond this point, strength remained stable, as the samples had already lost most of their structural integrity and exhibited significant cracking. Overall, as porosity increased, the mechanical properties declined, with a drastic reduction in strength observed in the MKPC-3 formulation. The compressive strength values obtained are consistent with those reported in previous studies on MPC-based foamed concretes formulated with H_2_O_2_ as a foaming agent, although using ADP instead of MKP [29].

## 4. Conclusions

This study demonstrates the feasibility of developing porous sustainable magnesium phosphate cement (sust-MKPC) using an industrial low-grade MgO (LG-MgO) by-product, potassium dihydrogen phosphate (MKP), and hydrogen peroxide (H_2_O_2_) as an air-entraining agent (AEA). This approach contributes to sustainability by reducing the extraction of pure MgO and the associated CO_2_ emissions. Structural characterization via X-ray diffraction (XRD) and Fourier-transformed infrared spectroscopy (FT-IR) identified K-struvite as the predominant phase in the MKPC matrix, alongside residual unreacted phases that serve as reinforcing agents. Increasing the H_2_O_2_ content led to higher porosity, reduced density, and consequently, decreased thermal conductivity and thermal diffusivity, contributing to improved thermal insulation and lightness. Furthermore, thermal inertia improved with the increasing AEA content, due to the associated reduction in thermal conductivity. However, these benefits were accompanied by a significant deterioration in mechanical performance, notably in terms of strength and stiffness (modulus of elasticity), especially beyond 3 wt.% H_2_O_2_. Densities below 1 g·cm^−3^ are achieved, resulting in thermal conductivities close to 0.1 W·m^−1^·K^−1^, through a porosity higher than 50%, while maintaining the compressive strength above 1 MPa. Accordingly, the sust-MKPC formulations with H_2_O_2_ contents exceeding 3 wt.% can be considered lightweight materials with promising thermal insulation properties. Compared to conventional insulating materials used in external thermal insulation composite systems (ETICS), such as organic foams and mineral wool, these formulations offer potential advantages for enhancing energy efficiency in building applications. Importantly, the developed sust-MKPC is not intended to replace conventional heating or cooling systems, nor existing insulation materials, but rather to serve as a complementary passive thermal buffer, mitigating indoor thermal fluctuations. Overall, this study highlights the potential of eco-friendly sust-MKPC formulations for integration into passive conditioning systems, contributing to energy savings in climate control applications.

## Figures and Tables

**Table 1:** Mix design of sust-MKPC with varying H_2_O_2_ percentages.

Sample	LG-MgO (%)	MKP (%)	Water (%)	H_2_O_2_ (%)	L/S
MKPC-0	60.0	40.0	34.0	-	0.34
MKPC-1	60.0	40.0	33.6	0.3	0.34
MKPC-2	60.0	40.0	33.2	0.7	0.34
MKPC-3	60.0	40.0	32.9	1.0	0.34
MKPC-5	60.0	40.0	32.2	1.7	0.34
MKPC-7.5	60.0	40.0	31.4	2.5	0.34
MKPC-10	60.0	40.0	30.5	3.4	0.34

**Table 2:** Chemical composition of the LG-MgO by means of XRF.

Oxide	MgO	CaO	SO_3_	SiO_2_	Fe_2_O_3_	Al_2_O_3_	LOI ^1^
**wt.%**	70.2	7.6	6.6	2.6	2.5	0.4	10.1

^1^ Loss on ignition at 1000 °C.

## Data Availability

The data supporting this study are available from the corresponding author upon request.

## Abbreviations

The following abbreviations are used in this manuscript:

MKPC	Magnesium potassium phosphate cement
CBPC	Chemically bonded phosphate ceramic
OPC	Ordinary Portland cement
LG-MgO	Low-grade magnesium oxide
AEA	Air-entraining agent
Sust-MKPC	Sustainable MKPC
GHG	Greenhouse gases
MPC	Magnesium phosphate cement
HVAC	Heating, ventilating, and air conditioning
PSD	Particle size distribution
XRF	X-ray fluorescence
XRD	X-ray diffraction (XRD)
FT-IR	Fourier-transformed infrared spectroscopy
ATR	Attenuated total reflectance
LOI	Loss on ignition
MOE	Modulus of elasticity
